# Silicon-Based Zipper Photonic Crystal Cavity Optomechanical System for Accelerometers

**DOI:** 10.3390/mi14101870

**Published:** 2023-09-29

**Authors:** Hongyu Tan, Debin Pan, Chensheng Wang, Yuan Yao

**Affiliations:** Wuhan National Lab for Optoelectronics, Huazhong Institute of Electro-Optics, Wuhan 430074, China; thy_oei@163.com (H.T.);

**Keywords:** accelerometer, zipper cavity, photonic crystal, high precision

## Abstract

The cavity optomechanical accelerometer based on photonic crystal microcavities combines mechanical resonators with high-quality factor photonic crystal cavities. The mechanical vibrator is sensitive to weak force/displacement in mechanical resonance modes, which can achieve extremely low noise levels and theoretically reach the standard qillatum noise limit. It is an important development direction for high-precision accelerometers. This article analyzes the principle and structural characteristics of a zipper type photonic crystal cavity optomechanical accelerometer, and designs a silicon-based zipper type photonic crystal cavity and mechanical vibrator structure applied to the accelerometer. The influence of the structural parameters of the zipper cavity on the optical Q factor was analyzed in detail. The resonant frequency of the optical cavity was controlled around 195 THz by adjusting the structural parameters, and the mechanical resonance characteristics of the mechanical vibrator and the optical cavity were analyzed. The effective mass of the optical cavity was 30 pg, and, with the addition of the mechanical vibrator, the effective mass was 3.1 ng. The optical mechanical coupling rate reached the GHz/nm level, providing guidance for the manufacturing and characterization of silicon-based zipper cavity accelerometers.

## 1. Introduction

As one of the basic inertial devices, accelerometers are widely used in military and civilian fields such as industrial automation, robotics, aerospace, automobiles, consumer electronics, and earthquake recording. There are already various accelerometers with different working principles, including piezoresistive [1,2,3,4], capacitive [5,6,7], resonant [8,9,10,11], optical [12,13,14], etc. Traditional piezoresistive and capacitive accelerometers use pressure or displacement caused by external acceleration to convert changes in electrical parameters through structural design to achieve acceleration measurements. Their structures are simple, and the manufacturing process is mature, but their sensitivities are not high. Resonant accelerometers characterize acceleration by detecting changes in resonant frequency caused by acceleration, and have high sensitivity, but the circuit design is relatively complex. The tunneling accelerometer detects acceleration by detecting tunnel current, which can achieve high accuracy but possesses a complex processing technology. The optical accelerometer utilizes a system that combines optical components with mechanical vibrators to characterize and detect changes in acceleration using optical parameters. It has advantages such as high accuracy and resistance to electromagnetic interference, but also has disadvantages such as poor temperature stability and processing difficulties.

In recent years, due to the sensitivity of mechanical vibrators in cavity optomechanical structures to weak forces/displacements in mechanical resonance modes [15], cavity optomechanical structures have rapidly developed in the field of high-precision sensing [16]. The cavity optomechanical structure is a structure that couples and exchanges momentum between electromagnetic radiation and mechanical vibrators by combining mechanical resonators with high-quality factor optical resonators [17]. Through linear and nonlinear optomechanical interactions in cavity optomechanical structures, quantum physics is connected to classical physics, and special physical phenomena such as optical springs [18], quantum ground state cooling [19], quantum entanglement [20], slow light generation, and quantum state transfer [21] have emerged, which have important scientific research significance in both fields. By utilizing these unique characteristics, the accelerometer with a cavity optomechanical structure can achieve extremely low noise levels, theoretically reaching the standard quantum noise limit. At the same time, the cavity optomechanical structure can also be integrated to achieve an accelerometer with a micro optomechanical structure.

Based on the advantages of the cavity optomechanical structure and the advantages and disadvantages of existing accelerometers, in order to meet the widespread military and civilian needs in the field of high-precision accelerometers, further research was conducted to achieve a mass-produced high-precision accelerometer based on a cavity optomechanical structure that has good electromagnetic interference resistance, simple processing technology, and can be integrated with current CMOS (Complex Metal Oxide Semiconductor) circuits. For the design method and simulation method, it is necessary to overcome the theoretical and technical difficulties in the testing of processing and manufacturing methods, acceleration optical mechanical coupling principles, and corresponding sensitivity, resolution, and other technical indicators, to promote the development of new acceleration sensing principles and new technological directions based on cavity optical mechanical structures.

This article is based on the working principle of a cavity optomechanical accelerometer, and designs and simulates a silicon-based zipper cavity and corresponding mechanical vibrators. The designed optical cavity is a zipper structure composed of silicon with air slits. The photonic crystal composed of periodically distributed air holes with the same shape on the silicon nanobeam binds the light field in the air slits. The designed silicon-based zipper cavity has a resonant frequency of 195 THz, corresponding to the operating wavelength in the C-band, with a maximum Q factor of 10^5^ orders of magnitude. At the same time, a detailed design was carried out on the structure of the mechanical vibrator, and the impact of different cantilever beam sizes on the overall resonance frequency and effective mass was analyzed. The maximum effective mass can reach 5 ng. Finally, the impact of effective mass and Q factor on measured acceleration was analyzed, laying a theoretical foundation for subsequent sensor development.

## 2. Structure Design

**Figure 1:**
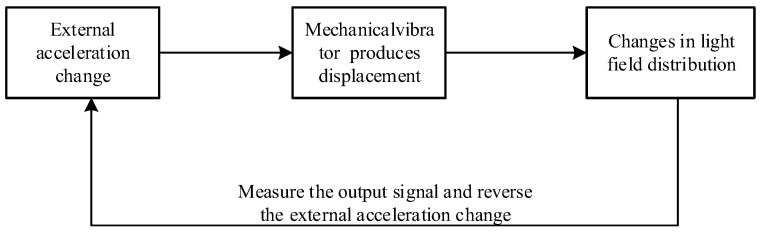
Schematic diagram of a typical cavity optomechanical accelerometer.

A typical cavity optomechanical system consists of two parts: a mechanical vibrator and an optical cavity. When acceleration is generated, the mechanical vibrator is affected by the acceleration to form a mechanical vibration mode and generate displacement. The coupled optical cavity generates changes in optical power due to the displacement of the mechanical vibrator. By detecting the output optical power, the measurement of acceleration can be completed, as shown in Figure 1. The optical cavity structure determines the distribution of the light field, and the mechanical vibrator determines the displacement caused by acceleration, which is an important condition for controlling the resolution of acceleration. Below, we will design and analyze the two separately.

### 2.1. Silicon-Based Zipper Photonic Crystal Cavity

A periodically arranged optical scale structure composed of dielectrics with different dielectric constants will modulate the electromagnetic waves transmitted within it, forming a photonic band structure. Photonic bandgap (PBG), also known as photonic bandgap, may occur in photonic band structures. When the electromagnetic wavelength meets the bandgap condition, it cannot propagate in the material. The periodic dielectric structure with a photonic bandgap is called photonic crystal [22].

According to the spatial distribution of photonic crystals, they can be divided into one-dimensional, two-dimensional, and three-dimensional photonic crystals. Firstly, periodic air holes and high-refractive index materials are used to form bandgap conditions. Materials such as silicon, silicon nitride, gallium arsenide, silicon oxide, etc. can be used, and then air holes or air slits are used to break the overall periodicity of the cladding, forming defect energy levels. Electromagnetic waves that meet a specific frequency are bound to the defect area due to their inability to enter the photonic crystal structure of the cladding.

The photonic crystal zipper cavity structure designed in this article for accelerometers is fabricated on silicon SOI (Silicon On Insulator) wafers on an insulator, which is well compatible with existing silicon-based technology systems and is a mature technology, greatly reducing manufacturing difficulties.

**Figure 2:**
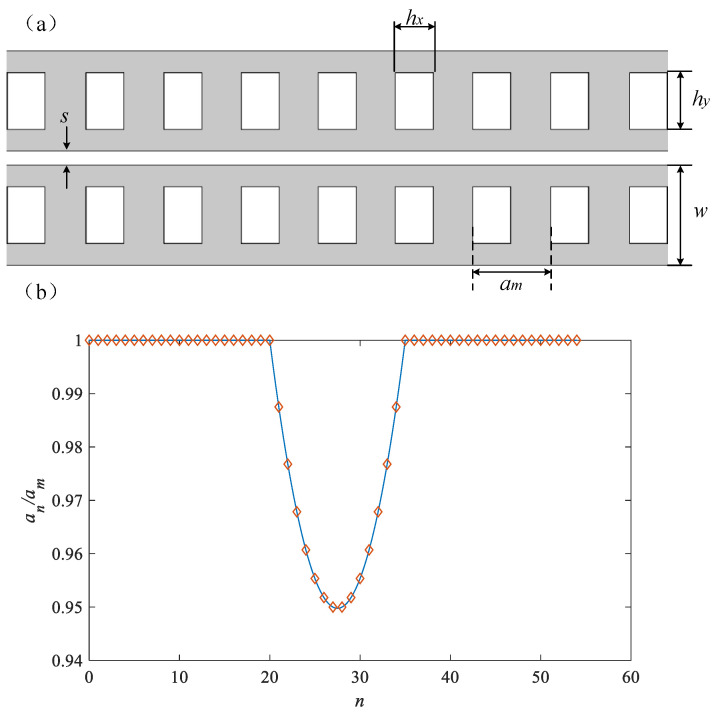
(**a**) Structure diagram of zipper cavity; (**b**) air hole spacing distribution diagram.

Figure 2a shows the structural parameters of the zipper cavity, which represent the air slit width (*s*), lattice constant (*a_m_*), nanobeam width (*w*), and axial and transverse air hole lengths *h_x_* and *h_y_*, respectively. We plotted the local lattice period in Figure 2b, defined as the relationship between an = x (n + 1) − x (n) and the number of pore intervals n along the length of the cavity. The length of the nanobeam is determined by the number of pore cycles in the cavity, and for standard structures, the number of pore cycles is *n* = 55. The defect region in the structure studied in this article consists of the lattice constants of a linear pore array near the center of the cavity changing along a quadratic function. We use a cavity defect area composed of 15 holes in the center, and the lattice period changes from the nominal value (*a_m_*) of the external reflection area in a quadratic function to 95% of the nominal value at the center of the defect area, as shown in Figure 2b. The diamond grid points represent the spacing data of each of the two air holes, and the ordinate is the ratio of spacing to standard spacing *a_m_*. It can be seen that the photonic crystal structure is composed of periodic air holes with a spacing of *a_m_* at both ends of the zipper cavity, making it impossible for light within the photonic bandgap range to propagate on both sides of the cavity. However, the air holes with a decreasing quadratic distribution of spacing in the middle break the periodicity and form defect regions, limiting the light within the photonic bandgap range to propagate at the center. The solid red line represents a function graph of the distribution, with constants at both ends and a piecewise function of a quadratic function in the middle, with zero points spaced at the center.

The design changes of the zipper cavity are centered around a “nominal” structure with the following (standardized) dimensions, as shown in Table 1: (1) beam width *w/a_m_* = 700/600, (2) beam thickness, *t/a_m_* = 220/600, (3) axial hole length, *h_x_/a_m_* = 214/600, (4) axial hole width, *h_y_/a_m_* = 400/600, and (5) air hole gap, *s/a_m_* = 60/600, all in nm, where *a_m_* is the lattice periodicity in the cavity reflection part.

### 2.2. Mechanical Vibrator

The basic unit of a mechanical vibrator is mainly composed of a mass block and a vibrating beam. The function of the beam is to suspend the mechanical vibrator in the air, which can vibrate under external forces. When there is acceleration in the sensitive direction, the mechanical vibrator forms a corresponding mechanical vibration mode. At the same time, in order to interact between the mechanical and optical modes, it is necessary to design half of the nanobeam cavity of the zipper cavity on the mechanical vibrator and the other half as fixed. In this way, the vibration mode of the mechanical vibrator will cause changes in the optical cavity, manifested as changes in the light field.

**Figure 3:**
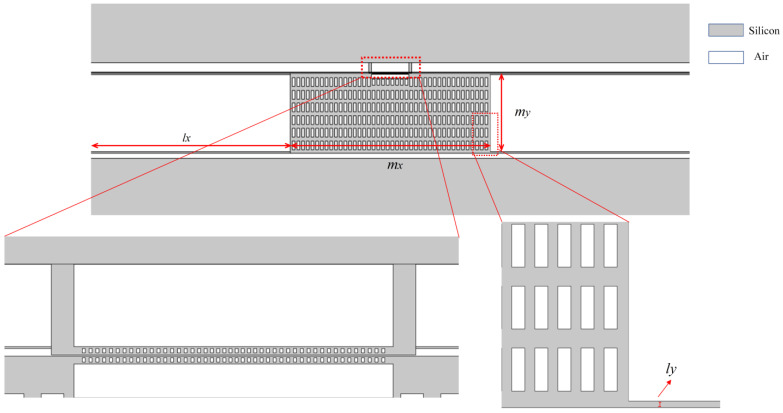
Structural parameters of zipper cavity optomechanical system.

The straight beam cavity optomechanical system consists of a large mass block, four straight beams connected to the top layer of the SOI wafer, a fixed block, and photonic crystal nanobeams loaded onto the mass block and fixed block, respectively. The overall thickness of this structure is 220 nm, and the nominal values of other structural parameters are: (1) the lateral length of the mass block *m_x_* = 150 µm; (2) the longitudinal length of the mass block *m_y_* = 60 µm; (3) the transverse length of the straight beam *l_x_* = 150 µm; (4) the longitudinal length of the straight beam *l_y_* = 60 µm; and (5) number of single side straight beams *N_l_* = 2. The overall structure is shown in Figure 3. The structural parameters of the mechanical vibrator are shown in Table 2.

## 3. Simulation Analysis

The modeling analysis mainly uses the finite element method [23,24] (FEM), which is a widely used and mature numerical calculation method. The finite element method mainly obtains the mode field characteristics of the optical cavity by solving the Helmholtz equation using an electromagnetic field module, while the mechanical mode characteristics can be analyzed by using a solid mechanics module. At the same time, the numerical solutions obtained from the simulation can be calculated through corresponding formulas to obtain corresponding optical and mechanical properties.

### 3.1. Optical Characteristics of Silicon Based Zipper Photonic Crystal Cavity

The magnitude of the optical Q factor and different optical modes can affect the optomechanical coupling rate, thereby affecting the sensitivity of the accelerometer. The optical mechanical coupling rate between the fundamental mode of the one-dimensional photonic crystal with symmetrical field strength distribution and the in-plane vibration mode of the mechanical vibrator is the highest [25], and the following parameters such as optical resonance frequency are all parameters of the fundamental mode.

**Figure 4:**
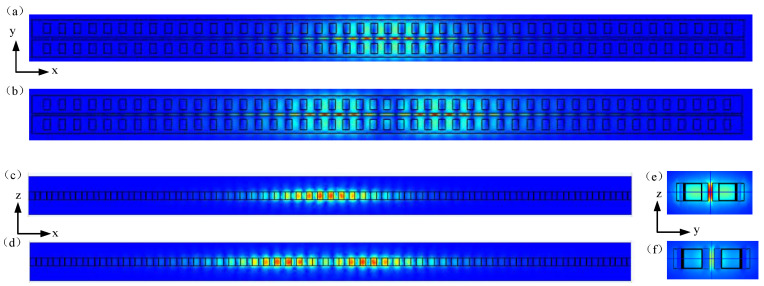
Distribution of zipper cavity simulation mode, electric field distribution in xy plane: (**a**) base mode; (**b**) second-order module. Electric field distribution in the xz plane: (**c**) fundamental mode; (**d**) second-order mode. The electric field distribution in the yz plane: (**e**) base mode; (**f**) second-order mode.

The coupling mode of the zipper cavity was obtained through simulation, which means that the electric field energy is mainly limited in the slit. Figure 4 shows the electric field distribution corresponding to the fundamental and second-order modes of the three cross-sections. Figure 4a,b shows the mode distribution in the xy plane, and it can clearly be seen that the electric field is mainly confined in the air slit. When the mechanical vibrator is affected by acceleration and vibrates, half of the nanobeams in the zipper cavity will generate corresponding displacements. The electric field confined in the slit is sensitive to this change, which is conducive to optomechanical coupling. Figure 4c,d shows the pattern distribution in the xz plane. It can be seen that each mode spot is spaced between two air holes in the x-direction. Figure 4e,f shows the mode distribution in the yz plane. The second order mode slit has less electric field energy in this plane because the center position of the zipper cavity was selected when cutting the plane, while the electric field in the x direction of the second order mode is mainly distributed on both sides of the center. The characteristic frequencies corresponding to the fundamental and second-order modes are 195.48 THz and 188.02 THz, respectively, with corresponding wavelengths of 1.53 μm and 1.59 μm.

**Figure 5:**
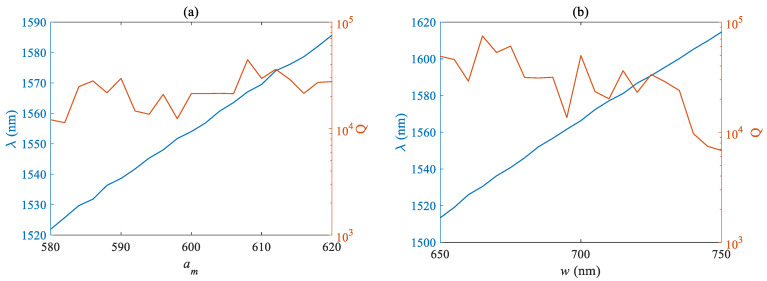
Relationship between Q factor and fundamental mode wavelength of (**a**) air hole period *a_m_* (**b**) and nanobeam width *w*.

The Q factor of an optical cavity is an important factor reflecting its quality. The influence of different structural parameters on the Q factor and wavelength was simulated and analyzed. The blue curve corresponds to the left axis, which is the wavelength curve in nm, while the orange curve corresponds to the right axis, which is the Q factor curve. Figure 5a,b respectively plots the curves of the Q factor and mode wavelength as a function of the air pore lattice period *a_m_* and nanobeam width w. Figure 5 shows that the required mode wavelength is positively related to the lattice period and nanobeam width, but its sensitivity to the nanobeam width w is lower than that of the lattice period *a_m_*. When the range of w variation is 100 nm, the wavelength fluctuation is only less than 60 nm, while a change of 40 nm in *a_m_* can shift the fundamental mode wavelength close to 65 nm. The tolerance of the lattice period needs to be more strictly controlled during actual manufacturing. The fluctuation of the Q factor is not very obvious, and it remains at around 4 × 10^4^ within the designed parameter variation range, with relatively large factors obtained at individual parameters. The Q factor showed a downward trend with the increase of nanobeam width, and the maximum value was about 8 × 10^4^.

**Figure 6:**
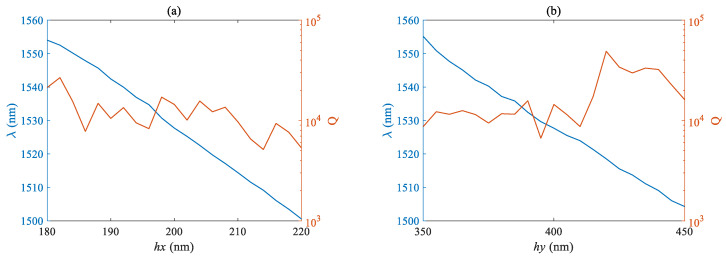
Relationship between Q value and fundamental mode wavelength of (**a**) the transverse length *h_x_* (**b**) and longitudinal length *h_y_*.

**Figure 7:**
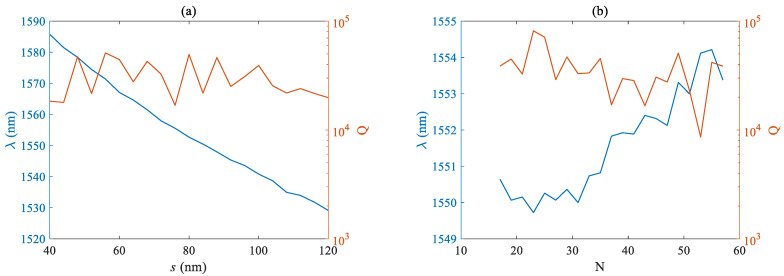
Relationship between Q factor and fundamental mode wavelength of (**a**) the slit width *s* (**b**) and number of holes *n*.

Figure 6a,b respectively plots the curves of the Q factor and mode wavelength as a function of the lateral length *h_x_* and longitudinal length *h_y_* of the air hole. The fundamental mode wavelength is negatively correlated with the size of the air hole, and the proportional coefficient is basically the same. However, due to the smaller nominal value of the lateral size, the same size fluctuation has a more significant impact on the wavelength and Q factor. The Q factor shows an overall downward trend with the increase of *h_x_*, and has a maximum value near 420 nm when *h_y_* is taken. Figure 7a,b respectively plots the curves of the Q factor and mode wavelength as a function of slit width s and pore number N. The fundamental mode wavelength is negatively correlated with the size of the air hole, and the overall Q factor shows a decreasing trend with the increase of the slit width s, but the fluctuation is not significant. From Figure 7b, it can be seen that the overall fundamental mode wavelength shows an upward trend with the increase of the number of holes N, but the fluctuation is not significant, at <5 nm, and the maximum Q factor is close to the order of 10^5^.

### 3.2. Mechanical Properties

**Figure 8:**
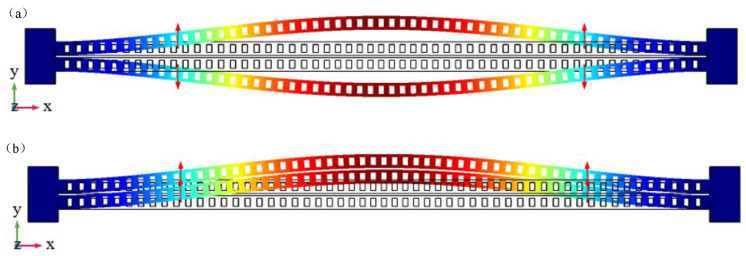
In-plane mechanical mode of xy plane of zipper cavity: (**a**) differential mode; (**b**) common mode.

At the same time, the mechanical mode of the zipper cavity was analyzed, and two fixed blocks were added at both ends. Fixed constraint conditions were used at the distal boundary of the fixed block, and the solid mechanics module in structural mechanics was used for simulation. Figure 8a,b respectively shows two xy in-plane mechanical modes. Figure 8a shows the differential mode, where the two sides of the zipper cavity are displaced in two directions, which has a more significant impact on the optical performance of the cavity. 

This mode is selected as the mode of optomechanical coupling, while the change in the same direction mode in Figure 8b is relatively small. The corresponding characteristic frequencies of the two modes are 4.82 MHz and 4.85 MHz, respectively, corresponding to an effective mass of 13.8 pg.

A further simulation analysis was conducted on the influence of different lengths on the resonant frequency of mechanical modes. Table 3 shows the differential modes at N = 47, 51, 53, and 55, with resonant frequencies of 6.56 MHz, 5.59 MHz, 5.18 MHz, and 4.82 MHz, respectively. The resonant frequency gradually decreases as the number of air holes or length of the zipper cavity increases.

After adding a mechanical vibrator, the mechanical vibrator and the zipper cavity form the optical mechanical system. The mechanical mode of the mechanical vibrator affects half of the optical cavities on the mechanical vibrator, driving changes in optical characteristics. The optical radiation pressure of the optical mode in turn heats or cools the mechanical mode of the mechanical vibrator. The mechanical modes of the mechanical vibrator mass block were also simulated and analyzed.

**Figure 9:**
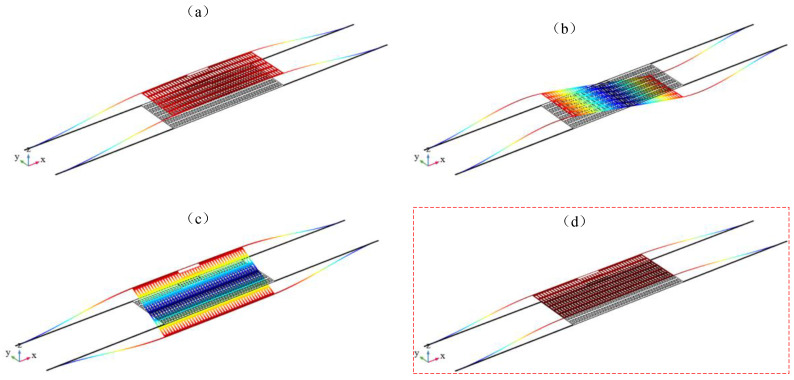
The first four mechanical modes of mechanical vibrator: (**a**) first order mode; (**b**) second order mode; (**c**) third order mode; (**d**) fourth order mode.

Figure 9 shows four mechanical modes of the mass block, with corresponding frequencies of 3.66 KHz, 13.04 KHz, 13.55 KHz, and 18.82 KHz, among which Figure 9a–c are all modes outside of the xy plane. The displacement of the mechanical vibrator is in the z direction, while the optical cavity is in the xy plane. These modes cannot achieve good optomechanical coupling. And Figure 9d shows the xy in-plane mode of the vibrator at this time, which is an in-plane vibration mode that vibrates along the direction of the detection acceleration. This mode will be “filtered” out by the light radiation pressure of a specific optical mode field and heated up for vibration, indicating that this mode has the highest optomechanical coupling rate. The main consideration is the relevant characteristics of this mode, and the relevant parameters that affect the mechanical mode characteristics of the mechanical vibrator include the size, quantity, and mass block size of the vibrating beam. Considering the actual manufacturing factors and simplifying the optimization process, the simulation here mainly focuses on the simulation analysis of the size and quantity of the vibrating beam.

**Figure 10:**
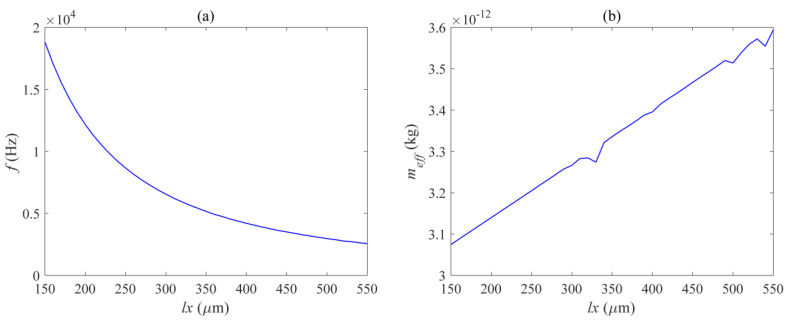
Relation diagram of (**a**) mechanical mode resonance frequency and (**b**) effective mass with beam length *l_x_*.

**Figure 11:**
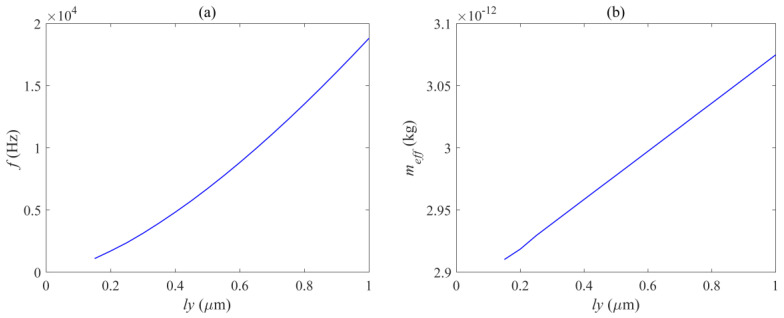
Relation diagram of (**a**) mechanical mode resonance frequency and (**b**) effective mass with beam width *l_y_*.

**Figure 12:**
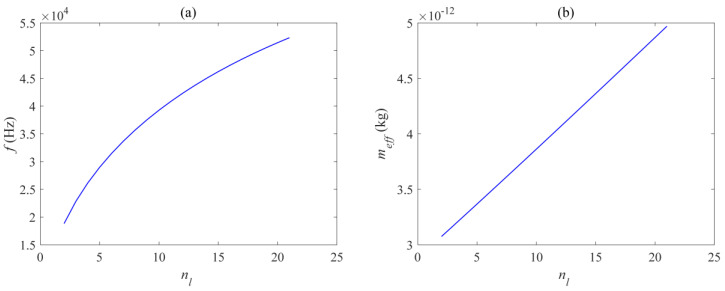
Relation diagram of (**a**) mechanical mode resonance frequency and (**b**) effective mass with beam number *N_l_*.

Figure 10, Figure 11 and Figure 12 respectively show the effects of different beam lengths *l_x_*, widths *l_y_*, and quantities *N_l_* on the in-plane mode resonance frequency and effective mass of the mechanical vibrator. The photomechanical coupling can be described by using a single acoustic mode of a mechanical resonator and its optomechanical coupling rate with light. At low frequencies, this effective response is equivalent to a harmonic response where the effective mass is less than the total mirror mass. The definition of effective mass *m_eff_* is as follows:

(1) $$m_{eff}=\int \rho (|Q(r){|}^{2}/\max(|Q(r){|}^{2}))dV$$

Among them, *ρ* is the density of the mechanical vibrator material, Q (r) is the displacement of each point of the mechanical vibrator, and max (Q (r)) is the maximum displacement of the mechanical vibrator in this mechanical mode.

Figure 10 shows the curve of the resonant frequency and effective modal mass as a function of the length of the vibrating beam. It can be seen that the relationship between the resonant frequency and the length of the vibrating beam is a first-class inverse proportional function, and decreases overall with the increase of *l_x_*. However, the decrease is rapid when *l_x_* is small, and as *l_x_* continues to increase to a certain value, the decrease rate of the resonant frequency slows down. Figure 11 shows the curve of the resonant frequency and effective modal mass as a function of the width of the vibrating beam, within the range of 0.15 μm to 1 μm, and the resonant frequency is positively correlated with *l_y_*. Figure 12 shows the curve of the resonant frequency and effective modal mass as a function of the width of the vibrating beam. The number of vibrating beams gradually increases from 2 to 21, and the resonant frequency is also positively correlated with the number of vibrating beams *N_l_.* From Figure 10b, Figure 11b, and Figure 12b, it can be seen that the effective mass is basically positively correlated with the size and quantity of the vibrating beam, and the transformation trend is close to a linear change. The main reason is that the increase in the size and quantity of the vibrating beam increases the overall displacement volume without changing the mode corresponding to the displacement distribution of the vibrating beam. The maximum effective mass is 5 ng, and the resonance frequency variation range is 18.8–52.3 KHz, which can meet various needs.

In the actual detection of accelerometers, mechanical sensitivity *χ*(*ω*) is an important standard for measuring the performance of an accelerometer, defined as the displacement of the mechanical vibrator caused by unit acceleration. The greater the mechanical sensitivity, the greater the displacement of the mechanical vibrator caused by unit acceleration. When other parameters are the same, the overall sensitivity of the accelerometer is higher. χ(ω) is given by the following equation [25]:

(2) $$\chi (\omega )=\frac{1}{{\omega_{m}}^{2}-\omega^{2}+i(\omega \omega_{m}/Q)}$$

**Figure 13:**
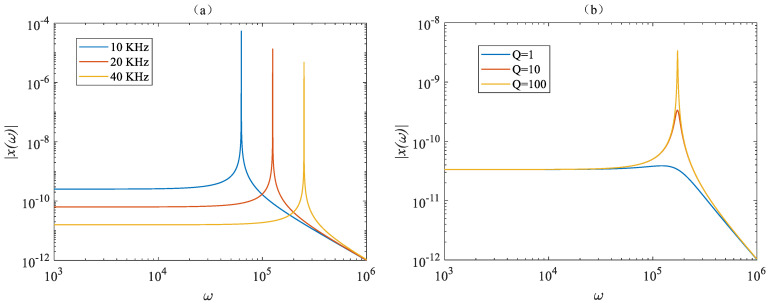
Relation curve between mechanical sensitivity and acceleration frequency for: (**a**) different mechanical frequencies; (**b**) different Q factors.

Among them, *ω_m_* is the resonant angular frequency of the mechanical vibrator, *ω* is the angular frequency of acceleration, *i* = √−1, and *Q* is the mechanical Q factor of the mechanical vibrator. Figure 13 simulates and analyzes the effects of different resonant frequencies and Q factors on mechanical sensitivity when other parameters are simultaneously present. From Figure 13a, it can be seen that, when the resonant frequency decreases, the overall mechanical sensitivity increases, but the flat frequency response bandwidth decreases. When *ω* << *ω_m_*, the mechanical vibrator has a flat response curve to AC acceleration, and when *ω* >> *ω_m_*, the mechanical sensitivity gradually decreases. Therefore, a compromised design must be made between bandwidth and sensitivity based on actual needs. And when *ω* = *ω_m_*, the mechanical sensitivity is the highest and positively correlates with the Q factor, so it is also necessary to increase the mechanical Q factor of the mechanical vibrator as much as possible to enhance mechanical sensitivity. At the same time, the displacement of the mechanical vibrator will cause changes in optical resonance frequency, and the optical mechanical coupling rate is defined as:

(3) $$g_{\mathrm{om}}=d\omega_{c}/dx$$

Among them, *ω_c_* is the angular frequency of the optical mode, while *x* is the displacement of the mechanical vibrator. In cavity optomechanical interaction, the optomechanical coupling index can be mainly divided into two parts. Firstly, the photoelastic effect caused by mechanical motion can change the refractive index of the medium, thereby changing the resonant frequency of the optical resonant cavity. This may be the dominant factor in some systems, such as micro disk structures. Secondly, the equivalent refractive index change caused by the moving dielectric boundary caused by mechanical motion leads to a change in the optical resonant frequency. In the F–P cavity, the second factor dominates. In the case of the commonly studied Fabry–Perot cavity, GOM = *ω_c_*/*L_c_*, where *L_c_* is approximately the physical length of the cavity. A similar relationship applies to echo wall structures, where the optical mechanical coupling is proportional to the reciprocal of the cavity radius (R), *g_om_* = *ω_c_*/R. Both devices utilize the radiation pressure or scattering force of light. In contrast, the zipper cavity uses gradient force operation, and its optomechanical coupling length can range from the optical wavelength *L_om_* to λ on the scale of c, where the length of optical mechanical coupling exponentially scales with the air slit *s*, *L_om_* ~ *λce^αs^*. Among them α is proportional to the refractive index comparison between the nanobeam forming the zipper cavity, and the surrounding cladding is directly proportional [26], which means that the optical mechanical coupling index *g_om_* of the zipper cavity can reach the GHz/nm level, achieving more effective optical mechanical coupling.

The previous analysis results were based on ideal situations, but in actual situations, due to the effect of gravity, there will be a certain height difference between the two ends of the photonic crystal air slots located on the mass block and the fixed block, which will affect the resonant frequency and optical Q factor of the photonic crystal. Therefore, it is necessary to consider the impact of height differences on the fundamental mode optical Q factor and conduct simulation analysis.

**Figure 14:**
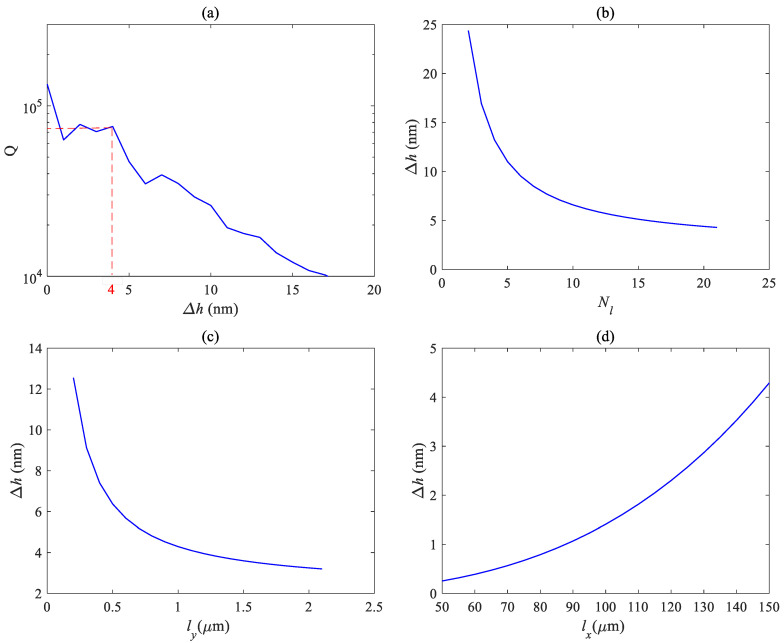
(**a**) Relation curve between mechanical sensitivity and acceleration frequency for (**a**) different mechanical frequencies; relationship between height difference and different beam parameters: (**b**) beam number *N_l_*; (**c**) beam width *l_y_*; and (**d**) beam length *l_x_*.

Figure 14 shows the influence of the height difference ∆*h* at both ends of the photonic crystal zipper cavity on Q and the influence of different beam parameters on height difference, respectively.

Figure 14a shows the variation of the Q factor with height difference ∆*h*. It can be seen that, when the height difference is within the range of 4 nm, the overall Q factor remains close to 10^5^, and after exceeding 15 nm, the Q factor has already decreased by an order of magnitude. So, it is necessary to maintain a height difference of less than 4 nm. Figure 14b–d shows the relationship between the height difference ∆*h* and the number of beams *N_l_*, beam width *l_x_*, and beam length *l_y_*, respectively. It can be seen that the height difference is negatively correlated with these parameters. That is, as the number of beams increases and the width of the beam increases, the traction force on the mass block increases, and the displacement of the vibrator affected by gravity decreases, resulting in a smaller height difference. As the beam becomes longer, the traction force on the central mass block becomes weaker and the height difference becomes greater. From the figure, it can be seen that the height difference can be controlled within 4 nm by increasing the number of beams, increasing the width of beams, and reducing the length of beams. Due to the fact that beam parameters can also affect resonant frequency, it is not only necessary to consider the height difference during actual manufacturing, but also to obtain appropriate parameters based on factors such as actual needs.

## 4. Conclusions

This article designs an optical mechanical system based on a silicon-based zipper photonic crystal cavity that can be used for accelerometers, which is compatible with existing processes. The overall structure of the system is analyzed in detail, and the influence of different structural parameters on the optical performance of the optical mechanical system is simulated and studied, such as mode wavelength and Q factor. The optimized zipper cavity parameters are as follows: beam width *w* = 700 nm, beam thickness *t =* 220 nm, axial hole length *h_x_* = 180 nm, axial hole width *h_y_ =* 400 nm, air hole gap *s* = 60 nm, and lattice constant *a_m_* = 608 nm. The Q factor is close to 10^5^, and the wavelength can be freely adjusted within the range of 1500–1600 nm by modifying the structural parameters. At the same time, the overall mechanical characteristics were analyzed, and a mechanical mode analysis was conducted on the zipper cavity and the entire system with mechanical vibrators. The basic structural parameters of the designed mechanical vibrator are as follows: the lateral length of the mass block *m_x_* = 150 µm; the longitudinal length of the mass block *m_y_* = 60 µm; the transverse length of the straight beam *l_x_* = 150 µm; the longitudinal length of the straight beam *l_y_* = 60 µm; and number of single side straight beams *N_l_* = 21. The influence of different structural dimensions on the mechanical mode frequency and effective mass was elucidated, with a maximum effective mass of 5 ng. At the same time, the influence of gravity on the vibrator can be significantly reduced, and the height of the mechanical vibrator descent affected by gravity will not exceed 4 nm. Finally, the influence of mechanical mode resonance frequency and mechanical Q factor on measuring AC acceleration was analyzed. A smaller resonance frequency and larger mechanical Q factor can achieve greater sensitivity.

For the next step in the research of the silicon-based zipper cavity optomechanical accelerometer, on the one hand, a more accurate theoretical model is adopted to analyze other performance parameters of the accelerometer system. In practical manufacturing, the presented structures are defined as a 4-inch SOI wafer and the thickness of the top silicon is 220 nm. After cleaning the SOI wafer, we spun a layer of 400 nm PMMA A4 photoresist, then the wafer was at 180 °C for two minutes. Electron beam lithography technology was used to write structural patterns, then developed, cleaned and spun dry. The designed structure was obtained by inductively coupled plasma etching on silicon. Acetone was used to remove the remaining photoresist, cleaned and spun dry. Finally, HF gas was used to release the silica under the top silicon, making the mechanical vibrator suspended. However, the specific process parameters and operations are still complex and require further research and experimentation. At the same time, stable devices have not been obtained, and targeted research on packaging processes is needed, to improve system reliability and reduce costs.

The designed silicon-based zipper cavity optomechanical system can be directly applied in the field of accelerometers. Small external acceleration disturbances can stimulate the mechanical mode of the mechanical vibrator, thereby changing the optical field changes of the zipper cavity. By coupling the laser into the zipper cavity through a tapered fiber and monitoring and analyzing the output light field characteristics, the acceleration information perpendicular to the axis of the zipper cavity can be obtained. With the development of technology, cavity optomechanical accelerometers have the advantages of high sensitivity, small size, low power consumption, and high resolution, which will drive them to become an important force in military and civilian development in the field of inertia.

## Figures and Tables

**Table 1:** Structural parameters of zipper cavity.

Parameters	*a_m_*	*w*	*t*	*h_x_*	*h_y_*	*s*
Value (nm)	600	700	220	214	400	60

**Table 2:** Structural parameters of mechanical vibrator.

Parameters	*m_x_*	*m_y_*	*t_m_*	*l_x_*	*l_y_*	*N_l_*
Value	150 µm	60 µm	220 µm	150 µm	1 µm	2

**Table 3:** Resonant frequency corresponding to two mechanical modes with different numbers of holes.

Number of Holes	47	49	51	53	55
In-plane differential mode (MHz)	6.56	6.05	5.59	5.18	4.82
In-plane common mode (MHz)	6.61	6.09	6.63	5.22	4.85

## Data Availability

The data presented in this study are available on request from the corresponding author. The data are not publicly available due to intellectual property rights.
